# Cerebral Autosomal Dominant Arteriopathy With Subcortical Infarcts and Leukoencephalopathy (CADASIL) Syndrome: A Case Report and Review of Literature

**DOI:** 10.7759/cureus.53469

**Published:** 2024-02-02

**Authors:** Cesar Gutierrez Gomez, Martin Daniel Alejandro Lopez Gonzalez, Adolfo Natanael Vazquez Tobias, José Guadalupe Rivera Chávez

**Affiliations:** 1 Internal Medicine, General Hospital of Leon, Leon, MEX; 2 Neurology, General Hospital of Leon, Leon, MEX; 3 Radiology, General Hospital of Leon, Leon, MEX

**Keywords:** cerebrovascular accident (stroke), cognitive impairment, notch3, migraine-type headache, cerebral autosomal dominant arteriopathy with subcortical infarcts and leukoencephalopathy (cadasil)

## Abstract

Cerebral autosomal dominant arteriopathy with subcortical infarcts and leukoencephalopathy (CADASIL) is an autosomal dominant genetic disorder of the small arteries that causes ischemic vascular events, subcortical dementia, behavioral changes, and migraine-like headaches. It is caused by a mutation in the NOTCH3 gene; this disease was first described in 1955 by van Bogaert.

We present a 29-year-old woman who presented to the neurology department. She has no history of chronic degenerative diseases. She has been complaining of migraine-like headaches for the past six months. She has cognitive impairment with arithmetic and executive function deficits on neurological examination. Blood biometry and blood chemistry are within normal parameters in her laboratory studies. A viral panel and immunological profile were also performed and were not reactive. A lumbar puncture was performed, and the composition of the cerebrospinal fluid was within normal limits. An MRI was performed, which showed bilateral and symmetric white matter hyperintensities consistent with CADASIL syndrome.

There is no specific treatment. Management of these patients is based on symptom control. Neurological sequelae have an important impact on the quality of life and mortality of these patients. For this reason, pharmacological preventive therapies have been sought with controversial evidence.

## Introduction

Cerebral autosomal dominant arteriopathy with subcortical infarcts and leukoencephalopathy (CADASIL) is an autosomal dominant genetic disorder of the small arteries that causes ischemic vascular events, subcortical dementia, behavioral changes, and migraine-like headaches. It is caused by a mutation in the NOTCH3 gene [1].

The disease was first described in 1955 by van Bogaert, who reported the diagnosis of "Binswagner's disease" in two sisters. This was a rapidly progressive vascular dementia characterized by hypertension, gait disturbance, and progressive cognitive impairment [2]. In 1976, however, a case of a 50-year-old man with multiple lacunar infarcts and extensive leukoencephalopathy was reported and initially classified as "Binswagner's disease"; however, the absence of arterial hypertension was noted as an atypical presentation and underwent genetic study. As a result of this genetic mapping, chromosome 19 was mapped. This is where the alteration in the NOTCH3 gene was first described [3].

In Mexico, vascular dementia is the most common cause of cognitive impairment in the adult population. Its incidence is 14.6 per 1,000 people per year. The prevalence is lower in women than men in the population under 89 years [4].

## Case presentation

**Figure 1:**
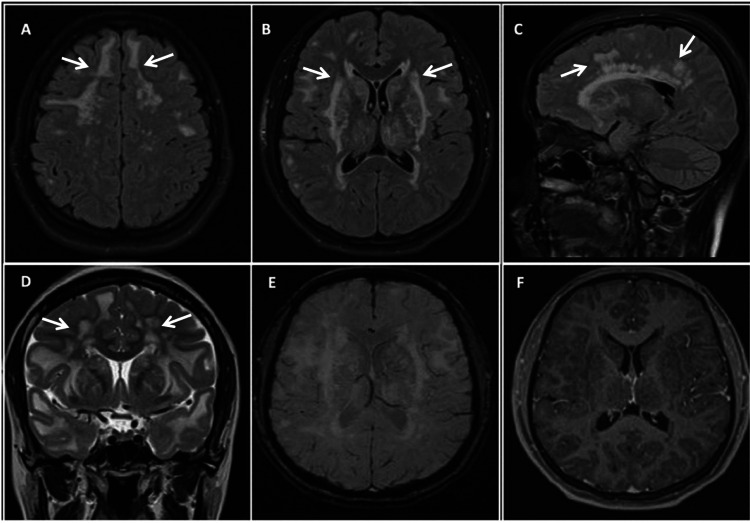
Brain MRI showing axial FLAIR (a and b), sagittal FLAIR (c), and coronal T2 (d) images showing white matter bilateral and symmetrical hyperintensities (white arrows) with a periventricular and subcortical distribution affecting the frontal, anterior temporal, parietal, and external capsule white matter. Axial SWI (e) shows no blooming artifact (microbleed). Post-gadolinium T1 (f) shows no abnormal enhancement MRI: magnetic resonance imaging, FLAIR: fluid-attenuated inversion recovery, SWI: susceptibility weighted imaging

A 29-year-old woman presents to the neurology department. She has no history of chronic degenerative diseases. For six months, she has complained of headaches, pressing type, intensity 8/10 on the visual analog scale, accompanied by photophobia, phonophobia, nausea, and vomiting, aggravated by the Valsalva maneuver, and partially relieved by aspirin. In the neurological examination, she has cognitive impairment with a score of 20 on the Montreal Cognitive Assessment Test, showing failure in calculation and executive functions. In her laboratory tests, the blood biometry shows a white blood cell count of 6,950 per mm3, a hemoglobin level of 14.9 g/dL, and 167,000 platelets per microliter of blood. The blood chemistry results are as follows: glucose 81 mg/dL, urea 26 mg/dL, blood urea nitrogen 12.1 mg/dL, creatinine 0.7 mg/dL, serum calcium 8.8 mg/dL, serum chlorine 110 mol/L, and serum potassium 4 mmol/L. A viral panel and immunological tests, including anti-dsDNA, rheumatoid factor, and anticardiolipin, were all negative. A lumbar puncture was performed. CSF protein and glucose levels were normal with a negative microbiological culture. An MRI was performed (Figure 1), and as it suggested CADASIL disease, testing for a mutation in the NOTCH3 gene was requested, and the diagnosis was confirmed.

## Discussion

Pathophysiology

CADASIL syndrome can be caused by a mutation in more than 150 domains of the NOTCH3 gene, which is expressed predominantly in the vascular smooth muscle of the small caliber arteries of the central nervous system. Mutations alter the inhibition and expression of smooth muscle cells, which is consistent with our distinctive MRI findings. This leads to arterial elongation, asymmetry, dilation, and constriction of the arteries, resulting in impaired blood flow [1]. The vascular involvement in CADASIL affects the white and gray matter, resulting in a thickening of the vessel walls and a narrowing of the lumen. Degeneration of the tunica media with granular deposits and immunoreactivity has also been described [5]. It is hypothesized that a mutation in NOTCH3 causes increased rather than decreased NOTCH function. The proposed criteria for recognizing pathognomonic changes due to NOTCH3 mutations are clinical data typical of CADASIL syndrome, diffuse white matter hyperintensities, mutations that are not considered polymorphisms, and periarteriolar granular deposits.

Although CADASIL is usually caused by a heterozygous mutation in NOTCH3, cases with homozygous mutations have been demonstrated, and a spectrum of severe clinical manifestations has been described [6]. The difference in clinical manifestations and disease course despite having the same mutation in the NOTCH3 gene suggests that there are environmental factors that may influence the disease. Tikka et al. (2009) published an article suggesting the existence of different phenotypes on the basis of a case report of monozygotic twins with clinical manifestations 14 years apart [7].

In addition, the presence of cardiovascular risk factors may also have an impact on the severity of disease presentation. Adib-Samii et al. (2010) analyzed 200 symptomatic patients in the United Kingdom and found that hypertension and smoking were associated with an increased risk of cerebrovascular event debut, with ORs of 2.57 and 1.07, respectively [8].

Clinical manifestations

Migraine with aura is the most common manifestation in CADASIL patients. It has been estimated that its prevalence varies from 20% to 40% of all patients, with an average age of presentation of 25 years in women and 30 to 35 years in men, with an average duration of 72 hours. Some patients, like the general population, relate headache episodes to stress, fatigue, and menstrual cycle symptoms [5]. Dischgans et al. (2008) described a series of patients in 50% of patients with CADASIL who experienced a decrease in headache frequency after their first cerebrovascular event [9].

**Figure 2:**
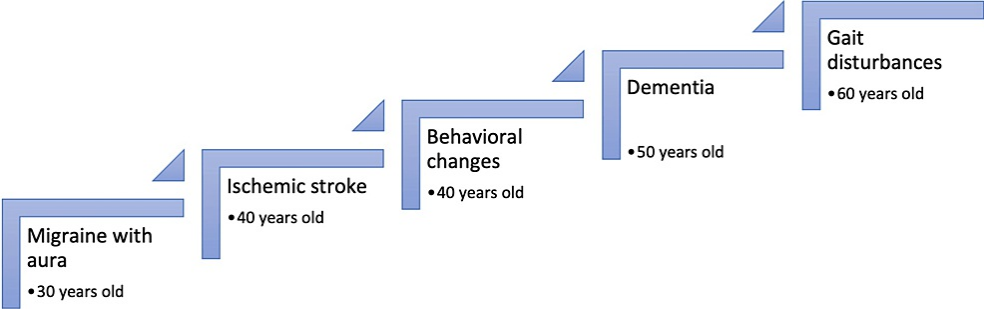
Age of appearance of clinical manifestations Image Credit: Authors

Stroke or transient ischemic events have been described as the most common manifestation of CADASIL, occurring in 60% to 85% of people with the disease. The average age of onset is 49 years, with a range of 20 years to 70 years of age [10]. The most common location is subcortical, so they have the same clinical manifestations as lacunar syndromes, including pure motor deficits, ataxic hemiparesis, and combined motor and sensory deficits. Most patients have recurrent events leading to rapidly progressive cognitive impairment, gait disturbances, and even incontinence [11]. The order of the appearance of symptoms is arranged chronologically in Figure 2.

Psychiatric manifestations have also been described, mostly classified as severe depression alternating with manic episodes, so these patients are usually diagnosed and treated as bipolar until imaging studies are performed due to cognitive impairment and the characteristic abnormalities of CADASIL are observed on MRI.

Cognitive impairment is the second most common clinical manifestation of CADASIL. The earliest signs of cognitive impairment are changes in executive function and speed of information processing, as measured by tests such as the Wisconsin Chart [10]. Cognitive impairment becomes more severe over time, affecting instrumental, verbal, or visual memory and progressing to dementia after several years [10]. Other less common clinical manifestations include seizures, in 5-10% of cases; intracerebral hemorrhages, which have also been associated with episodes of hypertensive crisis; and symptoms of parkinsonism [10].

Diagnosis

The original diagnostic criteria were proposed by Davouis in 1998; he was the first to suggest that this disease existed when it was not yet recognized by any physician. However, because these criteria were strict but had low sensitivity, they could only be applied to people with typical clinical manifestations. To avoid underdiagnosing this disease in Japan in 2017, Mizuno et al. (2020) postulated new criteria, which had a sensitivity of 97% (Table 1) [5].

**Table 1:** New diagnostic criteria for CADASIL GOM: granular osmiophilic material, CADASIL: cerebral autosomal dominant arteriopathy with subcortical infarcts and leukoencephalopathy, CT: computed tomography, MRI: magnetic resonance imaging [4]

Clinical criteria
1. Age of onset (clinical symptoms or white matter lesions) ≤55 years
2. At least two of the following
a. Pseudobulbar palsy or signs of dementia
b. A stroke-like episode with a focal neurological deficit
c. Behavioral changes
d. Migraine headache
3. Autosomal dominant heredity
4. White matter lesions involving the anterior temporal pole on magnetic resonance imaging or computed tomography scan
5. Leukodystrophy excluded
Genetic criteria
NOTCH3 mutations are located in exons 2 to 24. They cause a gain or loss of cysteine residues in the epidermal growth factor-like repeat domain
Pathologic criteria
GOM detected by electron microscopy is the pathologic feature of CADASIL. The immunostaining of the extracellular domain of NOTCH3 is also useful
Definitive
CADASIL is definitive when the following criteria are all met in the individual case. (1) White matter lesions that are demonstrated by CT or MRI. (2) Clinical criteria are present. (3) Genetic or pathology criteria
Probable CADASIL
CADASIL is probable when the individual has fulfillment of clinical criteria 1 to 5
Possible
CADASIL is possible when the individual has abnormal white matter lesions (Fazekas grade ≥2) and meets any of the following criteria. (1) ≤55 years of age. (2) At least one of clinical criterion 2 symptoms

Neuroimaging

The key to suspecting and diagnosing CADASIL is the evaluation of imaging studies. While a CT scan may be performed initially, it is the MRI signs that are the most sensitive and can lead to a diagnosis. These include white matter hyperintensities, lacunar infarcts, and cerebral microbleeds [11,12].

White matter hyperintensities seen on T2-weighted and FLAIR images on MRI often appear before symptoms appear. They are apparent areas of high signal (usually periventricular and in the deep white matter). They are often bilateral and symmetrical. The regions most commonly involved are the frontal, parietal, anterior temporal, and external capsules. The latter is useful for differentiation from other forms of small vessel disease [10,11]. Areas such as the occipital white matter, basal ganglia, thalamus, and internal capsule are less commonly involved. Involvement of the corpus callosum is extremely rare. When present, multiple sclerosis is more likely than CADASIL [13].

Lacunar infarcts are lesions of white matter that are larger than 2 mm and have a signal intensity in the cerebrospinal fluid. In CADASIL, they usually affect the semi-oval center, thalamus, basal ganglia, and pons. The number of lacunar infarcts is correlated with the degree of cognitive impairment in the patient [14].

Microbleeds can be detected using susceptibility sequences. These sequences cause small areas of T2 signal loss to bloom, magnifying the image and making it more visible. Cortical and subcortical areas, white matter, the thalamus, and the pons are typical sites of microbleeds in CADASIL [15].

Histopathological alterations

Although the manifestations are predominantly neurological, arteriopathy can be seen in the muscle, peripheral nerves, skin, liver, kidney, heart, and intestine. Because the disease affects not only skin vessels but also those of other organs, immunohistochemical studies of skin biopsies have been performed, but their sensitivity varies [16].

In the autopsies of patients with CADASIL, a dilatation of the ventricular system with a widening of the periventricular spasms can be seen in the coronal sections. In addition, there is a patchy appearance with a yellowish colocalization and an increase in consistency. Microscopically, the small, penetrating cerebral and leptomeningeal areas are involved. There are clusters of electrodense granular material interspersed with vascular smooth muscle cells. These are detectable by electron microscopy. These granules measure 10 to 15 nm in diameter and form clusters that can exceed 1 mm in size. These granules are composed of eosinophilic material, as seen by hematoxylin and eosin staining. They do not contain amyloid material, as they are negative for Congo red staining. Weigert's and Van Gieson's elastic stains also show more pronounced degeneration of the tunica media [16].

Treatment

Migraine

A migraine-like attack should be treated with NSAIDs such as paracetamol, ibuprofen, naproxen, and diclofenac, combined with antiemetics such as metoclopramide and domperidone. Triptans may cause cerebral vasoconstriction and increase the risk of an ischemic event, and there is insufficient evidence to support their use. Their use should only be considered if the use of NSAIDs has been without adequate pain control [17].

Lifestyle changes should be considered. These include an appropriate diet, sleep hygiene, and the avoidance of stressful events, as these have been shown to be predisposing factors for migraine attacks. If symptom control is inadequate, the use of pharmacological prophylaxis for migraine may be considered. The use of beta-blockers such as flunarizine, amitriptyline, and topiramate has proven effective. However, because of their potential adverse effects on cognition and mood [17], they should be monitored.

In patients with concomitant epilepsy, the use of magnesium valproate, originally described as a second-line agent for primary migraine prophylaxis, is preferred. As well as using serotonin reuptake inhibitors in patients who already present with mood disorders associated with migraine [17].

The use of acetazolamide has been suggested as a prophylactic treatment for migraine. This has been specifically suggested for patients with epilepsy [18].

Cerebral Vascular Event

It is the most common clinical manifestation in patients with CADASIL, being present in 84% of cases, and is recurrent, being the main reason for the development of disability in patients. Its initial management should be the same as in the general population, with better results with the use of thrombolysis when criteria are met, compared to recanalization therapy [19].

As mentioned above, neurological sequelae have an important impact on the quality of life and mortality of these patients. For this reason, pharmacological preventive therapies have been sought. The use of aspirin has not shown efficacy. On the contrary, in the study by Oh et al. (2008), aspirin use as primary prophylaxis was associated with increased bleeding. However, its use at low doses (50-325 mg) as monotherapy or in combination with clopidogrel may be useful as secondary prophylaxis, according to guidelines for the management of cerebral vascular events [20].

The use of statins is controversial. Peters et al. (2007) conducted a study in which they enrolled 25 patients (16 women and nine men) diagnosed with CADASIL with no history of cerebral vascular events, no use of statins in the three months prior to enrolment, hypercholesterolemia defined as serum cholesterol concentration greater than 200 mg/dl, LDL levels greater than 120 mg/dl, and hypertension. They were treated for eight weeks with atorvastatin, 40 mg for the first four weeks and 80 mg for the next four weeks. None of the patients had a cerebral vascular event during the trial, and no difference in statin use was found in terms of dose; however, double-blind, randomized clinical trials are needed to rule out a beneficial effect of statin use [21].

Behavioral Changes

There are no studies that provide statistically significant evidence for the use of serotonin reuptake inhibitors in patients with CADASIL whose clinical presentation is a depressive episode, also referred to as "depression of vascular origin." Swenson et al. (2006) conducted a systematic review that did not find an association between the use of these drugs and the risk of adverse cardiovascular events. There are, however, case reports of their use in CADASIL patients with depressive symptoms [22].

Cognitive Impairment

The disability caused by cognitive impairment in CADASIL patients has been one of the main therapeutic targets to try to reduce the progression of these manifestations and improve quality of life. Dichgans et al. (2008) published a study in which a group of 168 patients with dementia of vascular origin, including CADASIL syndrome, half of whom were randomized to receive 10 mg of donepezil versus placebo for 18 weeks, with the primary objective of demonstrating cognitive improvement using the ADAS-cognitive scale. They did not find a statistically significant change in global cognition with the use of the drug, but they did show an improvement in the ability to perform some executive functions, such as processing speed and attention [9].

Galantamine, a cholinesterase inhibitor, was used in four patients in a study conducted by Posada-Rodríguez et al. (2008). They found that cognitive symptoms stabilized in two patients and improved in only one of them, but the small number of participants makes it statistically insignificant [23].

Life expectancy

Between March 2002 and 2004, Opherk et al. (2004) conducted a retrospective study in Germany. They included 411 patients with a genetic diagnosis of CADASIL. At baseline, 89% of patients had significant neurological sequelae characterized by tetraplegia or hemiparesis, 77% met diagnostic criteria for dementia, and 73% had dysarthria combined with dysphagia, so it's not surprising that 38% of deaths were due to pneumonia. It was also found that there were more deaths from sudden causes, such as arrhythmia and acute myocardial infarction, than in the general population. The average life expectancy in the study was 64 years for men and 70 years for women. However, those who lived to more than 70 years of age were totally dependent on their caregivers and were confined to beds [24].

## Conclusions

CADASIL syndrome is an autosomal dominant genetic disorder caused by a mutation in the NOTCH3 gene, which is expressed in vascular smooth muscle, resulting in systemic arteriopathy with impaired perfusion of multiple organs. Although changes in muscle, kidney, liver, and even heart biopsies have been described, the main manifestation of these patients is cerebral vascular events of an ischemic type, which may or may not be transient but lead to a rapidly progressive cognitive deficit, eventually causing dementia of vascular origin in young patients, with the consequent loss of quality of life and productive years from early stages of life. Diagnosis at an early stage is a priority, as observational studies report total disability in up to 89% of patients with this disease.

The average age of onset is 25 years in women and 30 years in men. The ischemic cerebrovascular event is the most common presentation. However, when questioned, up to 40% of patients report a history of severe headaches that do not fully respond to the use of NSAIDs and are even disabling, waking them up at night.

CADASIL should be included in the differential diagnosis of all young patients, male or female, who present with a cerebrovascular event at a young age despite other cardiovascular risk factors. It should also be considered a diagnostic option for patients being evaluated for migraine-like headaches with or without aura and poor response to treatment. The aim is to achieve an early diagnosis and to influence the neurological sequelae that are irreversible and lead to disability at an early age.

Although there is no definitive treatment for this condition, an increasing number of studies using different methodologies and different interventions have been carried out to improve the quality of life of patients with this diagnosis and prevent disease progression. However, the small number of patients with this disease is an important limitation for achieving statistical significance.
